# A multicellular brain spheroid model for studying the mechanisms and bioeffects of ultrasound-enhanced drug penetration beyond the blood‒brain barrier

**DOI:** 10.1038/s41598-023-50203-3

**Published:** 2024-01-22

**Authors:** Anurag N. Paranjape, Leonardo D’Aiuto, Wenxiao Zheng, Xucai Chen, Flordeliza S. Villanueva

**Affiliations:** 1 Center for Ultrasound Molecular Imaging and Therapeutics, University of Pittsburgh, Pittsburgh, PA USA; 2 Department of Medicine, University of Pittsburgh, Pittsburgh, PA USA; 3 Department of Psychiatry, University of Pittsburgh School of Medicine Western Psychiatric Institute and Clinic, Pittsburgh, PA USA; 4 Department of Health and Human Development, University of Pittsburgh, Pittsburgh, PA USA

**Keywords:** Biological models, Gene delivery, Cell biology, Neuroscience

## Abstract

The blood‒brain barrier (BBB) acts as a hindrance to drug therapy reaching the brain. With an increasing incidence of neurovascular diseases and brain cancer metastases, there is a need for an ideal in vitro model to develop novel methodologies for enhancing drug delivery to the brain. Here, we established a multicellular human brain spheroid model that mimics the BBB both architecturally and functionally. Within the spheroids, endothelial cells and pericytes localized to the periphery, while neurons, astrocytes, and microglia were distributed throughout. Ultrasound-targeted microbubble cavitation (UTMC) is a novel noninvasive technology for enhancing endothelial drug permeability. We utilized our three-dimensional (3D) model to study the feasibility and mechanisms regulating UTMC-induced hyperpermeability. UTMC caused a significant increase in the penetration of 10 kDa Texas red dextran (TRD) into the spheroids, 100 µm beyond the BBB, without compromising cell viability. This hyperpermeability was dependent on UTMC-induced calcium (Ca^2+^) influx and endothelial nitric oxide synthase (eNOS) activation. Our 3D brain spheroid model, with its intact and functional BBB, offers a valuable platform for studying the bioeffects of UTMC, including effects occurring spatially distant from the endothelial barrier.

## Introduction

The blood‒brain barrier (BBB) is a semipermeable membrane that exhibits high selectivity in allowing materials to pass from the blood to the brain parenchyma. The endothelial cells (ECs) in the brain capillaries form tight junctions that hold the adjacent ECs tightly, thereby greatly reducing paracellular permeability^1^. ECs also express efflux transporters that actively pump certain molecules back into the bloodstream^2^ and exhibit minimal pinocytic activity^3^. In addition, the presence of basement membranes, pericytes that wrap around the ECs, and astrocytic end-feet provide further structural support and reinforce the barrier function of the BBB^3^. Approximately 98% of small molecule drugs and nearly 100% of large therapeutic agents fail to cross the BBB^4^. Consequently, the BBB presents a significant obstacle in the delivery of drugs to the central nervous system (CNS) to treat various neurological disorders, such as Alzheimer’s disease, Huntington's disease, stroke, amyotrophic lateral sclerosis, gliomas and brain metastases.

To improve drug delivery to the brain, there are conventionally two broad strategies: modifying the drug itself to enhance its ability to cross the BBB or modifying the BBB to temporarily increase its permeability to standard drugs. Modification of drugs in some cases might reduce their biological activity, while invasive alteration of the BBB might cause adverse effects^5,6^.

In recent years, ultrasound (US) along with microbubbles has shown promise as a novel technology for delivering a drug or a gene locally to a specific region of the brain^7,8^. The microbubbles, which can also carry drugs, are gas-filled microspheres with a lipid shell, which cavitate (expand and contract) under the influence of US. US-targeted microbubble cavitation (UTMC) transiently increases endothelial barrier permeability^9,10^. There are significant benefits to using UTMC to open the BBB: this is a noninvasive technique, the BBB can be opened, and drugs can be delivered to a precise location in the brain by navigation of the US beam. BBB opening is reversible, and it has been reported that UTMC alone (even without drug delivery) can be helpful in treating some neurological disorders^11–13^.

Nevertheless, one of the potential secondary effects of applying UTMC to the brain under a given set of acoustic conditions is the occurrence of neuroinflammation^14^. Additionally, the mechanisms regulating UTMC-induced BBB opening and additional bioeffects are incompletely understood. There is a need for an ideal in vitro model that mimics the BBB and allows us to investigate the signaling mechanisms involved and to study the effects of UTMC on other brain cell types. While two-dimensional (2D) transwell systems offer a scalable and cost-effective alternative to study BBB permeability compared to in vivo models, they are limited in their ability to simulate cell‒cell interactions between and among different cell types and to explore the spatially remote effects of UTMC beyond the endothelial barrier. Furthermore, brain ECs, when grown in vitro, can lose key BBB properties^15,16^. Hence, improvement of in vitro BBB models to more accurately replicate the physiological conditions found in vivo and in a three-dimensional (3D) spatial context would greatly facilitate the ability to answer critical questions regarding the mechanisms and secondary effects of UTMC-induced BBB opening.

Human induced pluripotent stem cell (hiPSC)-derived 3D neuronal organoids have been developed to investigate viral infections of the CNS and neurological disorders^17,18^. In this study, we present a modified multicellular spheroid model comprising five important brain cell types (hiPSC-derived neurons and astrocytes cocultured with commercially procured primary human brain microvascular endothelial cells, pericytes and microglia) that enables free cell‒cell interactions. Our model mimics the BBB both architecturally and functionally, with ECs and pericytes organized at the periphery, while other cell types are distributed throughout the core of the spheroids. We show that UTMC enhances BBB permeability, as demonstrated by increased uptake of fluorescent dextrans. We report that UTMC-induced calcium (Ca^2+^) influx via mechanosensitive channels and subsequent activation of the endothelial nitric oxide synthase (eNOS) pathway in ECs regulate UTMC-induced hyperpermeability.

An ideal in vitro BBB model should offer researchers an accessible and cost-effective system that faithfully replicates physiological conditions. Key characteristics of such a model would include selective permeability, dynamic responses, presence of diverse transport mechanisms, representation of various brain cell types and their interactions, accurate simulation of drug transport for pharmaceutical studies, sensitivity to stimuli influencing barrier permeability, and compatibility with disease models for studying disorders. We have successfully created a 3D brain spheroid model featuring an intact and functional blood‒brain barrier. This model holds immense potential for conducting systematic investigations into the mechanisms governing BBB hyperpermeability and the bioeffects of innovative technologies such as UTMC. Importantly, it allows us to explore bioeffects that extend beyond the endothelial barrier and that may occur in regions that are spatially distant from the EC-microbubble cavitation interaction.

## Results

### Generation of multicellular 3D brain spheroids

**Figure 1:**
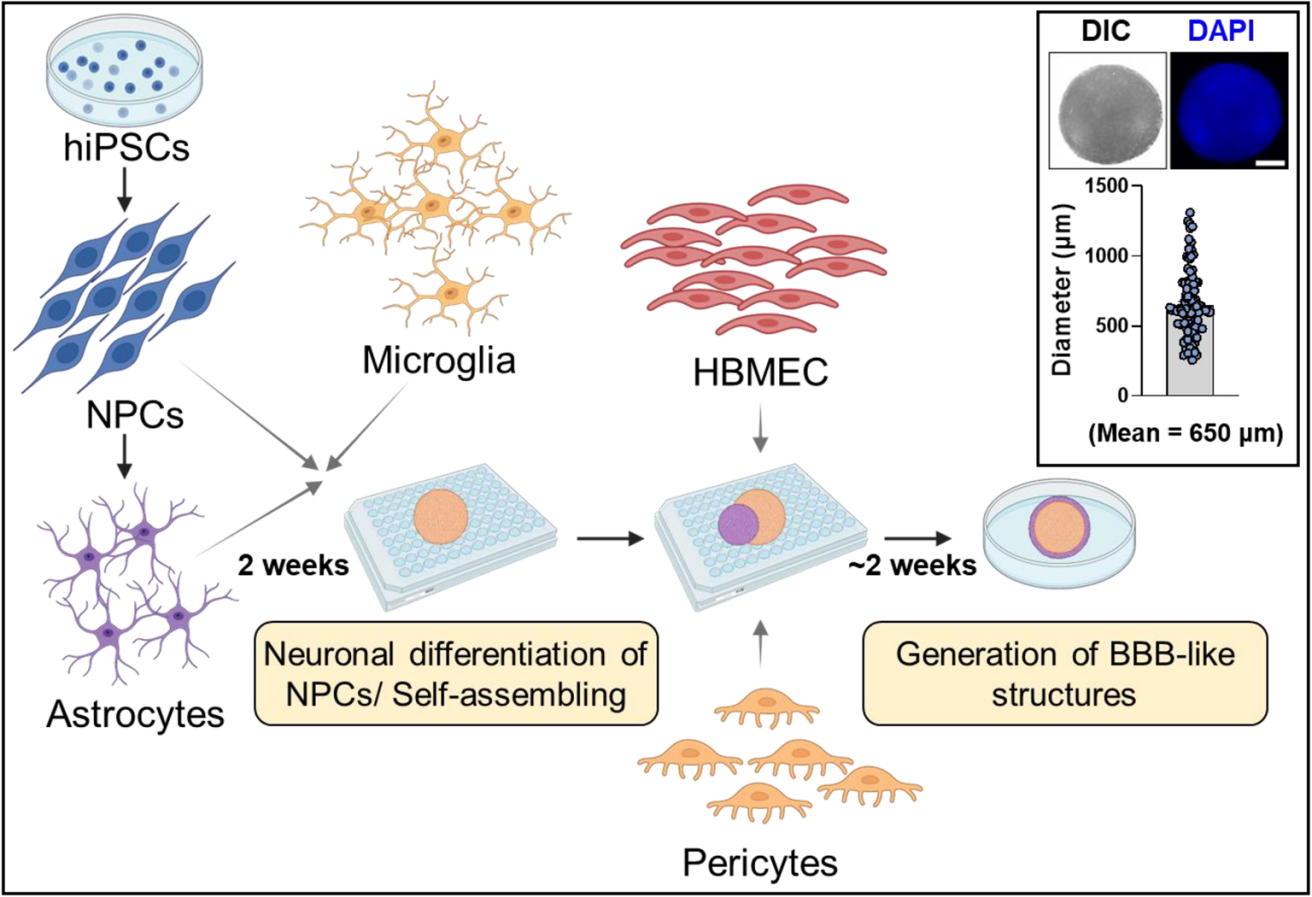
Schematic showing generation of the 3D brain spheroid model. Neural precursor cells (NPCs) and astrocytes generated from human induced pluripotent stem cells (hiPSCs) were mixed with human microglia and grown for 2 weeks. Later, human pericytes and human brain microvascular endothelial cells (HBMECs) were added and cultured (~ 2 weeks) until the spheroids were ready for experiments (detailed protocol provided under methods section). The inset shows the average diameter (650 µm) of spheroids used in the study (*n* = 100). The images show a typical spheroid. *DIC* differential interference contrast. Nuclei are counterstained with DAPI (blue). Scale bar = 100 µm.

To understand the mechanisms governing UTMC-induced hyperpermeability and its bioeffects, we established a 3D multicellular spheroid model comprising astrocytes and neurons (derived from hiPSCs), commercially procured primary human brain microvascular endothelial cells (HBMECs), pericytes and microglia (Fig. 1, refer to the methods section for more information). The initial step for the generation of the spheroids consisted of coculturing neural precursor cells (NPCs), astrocytes, and microglia cells in low-attachment 96-well plates, where the cells self-assembled and self-organized into spherical cellular aggregates (one spheroid/well). After two weeks of neuronal differentiation, spheroids were cocultured with HBMECs and pericytes. In approximately two weeks, HBMECs and pericytes merged and formed BBB-like structures on the surface of the spheroids (with an average diameter of 650 µm, Fig. 1- inset).

### The spheroids mimic BBB structure and function

**Figure 2:**
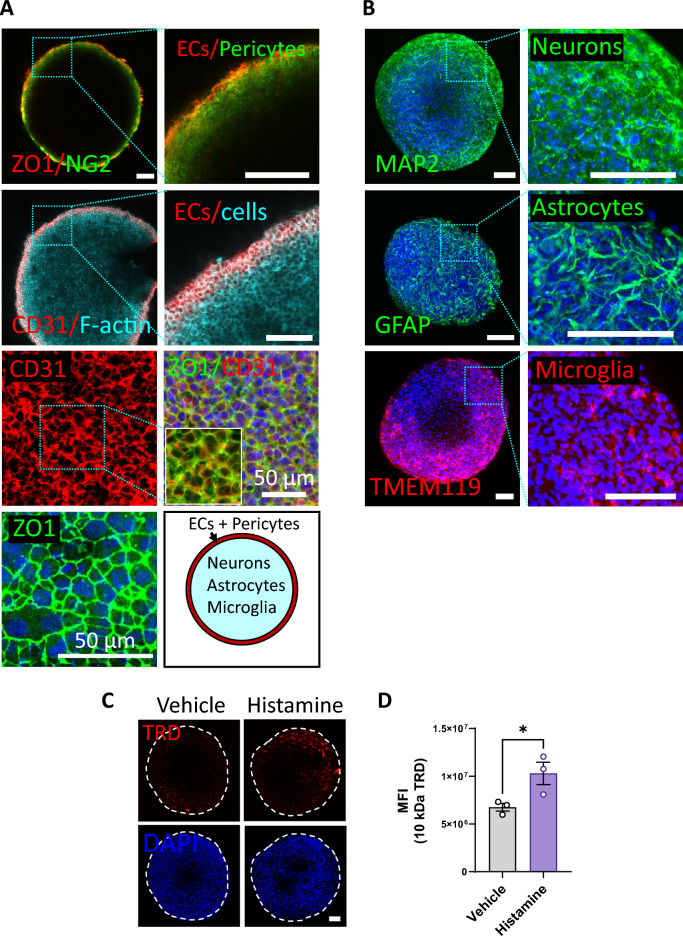
The spheroids mimic BBB structure and function. (**A**) The HBMECs (ZO1^+^, CD31^+^) and pericytes (NG2^+^) were found at the periphery of the spheroids. The HBMECs form a stable barrier, as shown by membranous expression of tight junction protein ZO1. (**B**) Neurons (MAP2^+^), astrocytes (GFAP^+^) and microglia (TMEM119^+^) are distributed throughout the spheroids (right panels in A and B show insets). (**C**) Representative images showing increased TRD uptake in spheroids treated with histamine (4.5 mM for 15 min). The spheroid boundary is shown with dashed white line. All images are maximum intensity Z-projections of the optical z-stacks. Nuclei are counterstained with DAPI (blue). Scale bar = 100 µm, unless mentioned otherwise. (**D**) Quantified TRD uptake (MFI: mean fluorescence intensity). Each dot represents one spheroid, from multiple experiments. The data represent mean ± S.E.M. Significance was calculated using unpaired parametric t-test (*p < 0.05).

To evaluate the spatial distribution of different brain cell types within the spheroids, we conducted immunofluorescence staining using specific markers for each cell type. Using confocal imaging, z-stacks of optical sections were taken from the spheroid surface to up to 200 µm depth into the spheroids (also refer to the methods section). Similar to previous reports^16,19,20^, the HBMECs (ZO1^+^, CD31^+^) and pericytes (NG2^+^) self-assembled at the outer surface of the spheroids (Fig. 2A). High magnification imaging at the surface revealed densely packed HBMECs exhibiting prominent membranous expression of the tight junction protein ZO1, which is a characteristic feature of a well-established barrier. The neurons (MAP2^+^), astrocytes (GFAP^+^), and microglia (TMEM119^+^) were distributed throughout the spheroid (Fig. 2B). This model, characterized by robust cell‒cell interactions, possessing an exterior endothelial barrier and interior brain parenchyma, is well suited for permeability studies. Additionally, this model serves as an ideal platform to investigate the modulation of signaling pathways and bioeffects on various brain cell types.

Histamine has been used to transiently open the BBB^20–22^. To assess the BBB functionality of our model, we tested the hyperpermeability induced by histamine. When spheroids were treated with histamine, there was a significant increase (52.4% increase, *p* < 0.05) in the amount of Texas red dextran (TRD) penetration into the spheroids (Fig. 2C), as shown by increased red fluorescence (maximum intensity Z-projections of the optical z-stacks taken from the top 20 µm to 200 µm depth within the spheroids) (Fig. 2D, Supplementary Fig. S1). The small quantities of TRD uptake in control spheroids may be attributed to sporadic openings in the endothelial barrier, an occasional occurrence in in vitro models.

### Ultrasound-targeted microbubble cavitation (UTMC) induces BBB spheroid hyperpermeability

**Figure 3:**
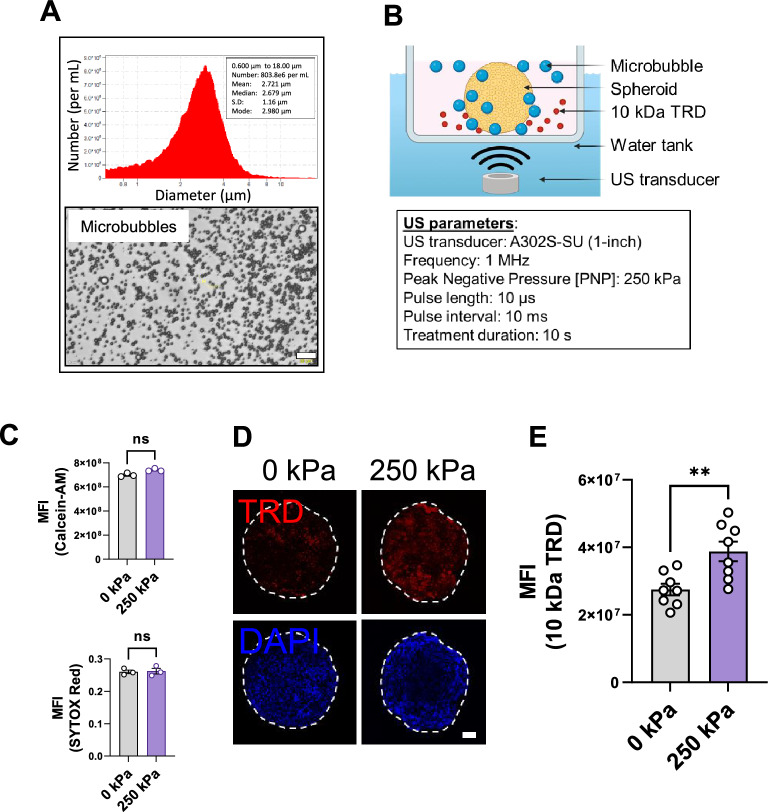
UTMC induced hyperpermeability in BBB spheroids. (**A**) Size distribution (upper panel) and appearance (lower panel) of microbubbles made in-house. (**B**) Schematic of the setup for UTMC treatment of spheroids (box shows the US parameters used). (**C**) Cell-viability measured 15 min post UTMC (upper panel: Calcein-AM, lower panel: SYTOX Red); each dot represents one spheroid, from multiple experiments (also refer to Supplementary Fig. S2). (**D**) Representative images showing TRD inside the spheroids. The spheroid boundary is shown with dashed white line. All images are maximum intensity Z-projections of the optical z-stacks. Nuclei were counterstained with DAPI (blue), scale bar = 100 µm. (**E**) Quantified TRD uptake. Each dot represents one spheroid, from multiple experiments. MFI: mean fluorescence intensity, the data represent mean ± S.E.M. Significance was calculated using unpaired parametric *t* test (*ns* not significant, **p < 0.01).

Microbubbles are small (around 1 to 10 µm diameter) gas (perfluorobutane) filled microspheres that cavitate in an US field (Fig. 3A). When microbubbles cavitate near the endothelial cell membrane of blood capillaries, they transiently open the endothelial barrier, thereby increasing drug extravasation into tissue^23^. As such, UTMC is a promising new tool to enhance drug delivery across the BBB. We tested whether UTMC increases permeability in our BBB spheroids. The spheroids were kept in a multiwell plate containing shallow media and microbubbles along with 10 kDa TRD, which was used as a model drug (Fig. 3B). Microscopically, the presence of microbubbles on the spheroids was confirmed (Supplementary Video S1). The plate was kept in a custom water tank that housed a submersible 1-inch single-element US transducer and treated with UTMC (frequency 1 MHz, with peak negative pressure [PNP] 250 kPa; pulse length 10 µs; pulse interval 10 ms; treatment duration 10 s; refer to Fig. 3B and methods section for more information). First, we confirmed that UTMC (250 kPa PNP) did not cause significant cell death using the cell viability dyes Calcein-AM and SYTOX Red (Fig. 3C, Supplementary Fig. S2A). Thereafter, we measured TRD uptake after UTMC. The spheroids treated with 250 kPa UTMC demonstrated a significant 53.1% increase in 10 kDa TRD uptake compared to the control group (*p* < 0.01) (Fig. 3D and 3E). This finding indicates that UTMC induces hyperpermeability in BBB spheroids, facilitating the uptake of 10 kDa TRD (no significant increase was observed when 70 kDa FITC-dextran was tested (Supplementary Fig. S4)). The spheroids remained viable for at least 60 min post UTMC (Supplementary Fig. S2B). We observed variation in TRD permeability in spheroids, which we hypothesize is mostly due to intrinsic structural heterogeneity of the spheroids. In spite of this variability, we observed significant and consistent enhancement of permeability upon UTMC.

### UTMC causes mechanosensitive channel-mediated Ca^2+^ influx, which is required for UTMC-induced hyperpermeability

Shear stress generated by cavitating microbubbles causes reversible cell membrane perforations (sonoporation), which contribute to increased Ca^2+^ influx^24–26^. Previous studies have shown that shear stress increases intracellular Ca^2+^ concentration through the influx of extracellular Ca^2+^, as this was dependent on Ca^2+^ in the media^27,28^.

**Figure 4:**
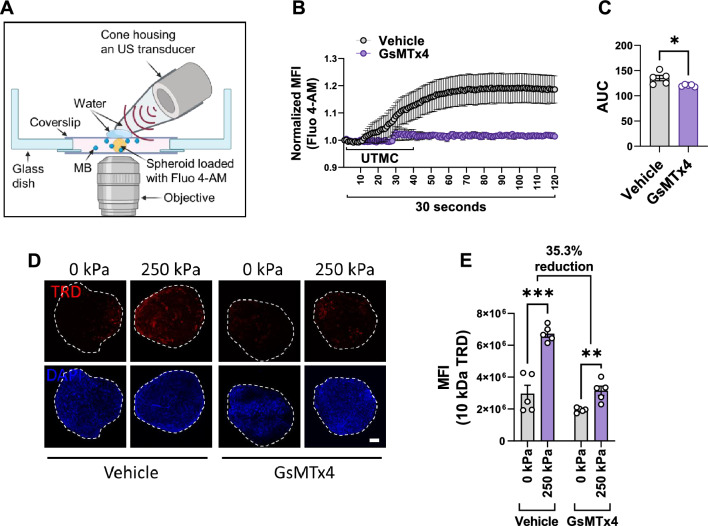
UTMC causes Ca^2+^ influx, via mechanosensitive channels, which is required for UTMC-induced hyperpermeability. (**A**) Schematic of the US setup for live-cell imaging of spheroids to assess Ca^2+^ influx (detailed information in the methods section). (**B**) Quantitation of UTMC-induced Ca^2+^ influx as measured by Fluo 4-AM fluorescence for up to 30 s (corresponds to 120 frames). UTMC was delivered from 1 to 10 s (corresponds to frames up to 40). (**C**) Area under the curve (AUC) of Fluo 4-AM fluorescence from panel (**B**) (*p < 0.05), each dot represents one spheroid, from multiple experiments. (**D**) Representative images showing TRD uptake in spheroids treated with mechanosensitive channel inhibitor GsMTx4 (1 µM for 30 min). Nuclei were counterstained with DAPI (blue). The spheroid boundary shown with dashed white line, scale bar = 100 µm. (**E**) Quantitation of TRD uptake. Each dot represents one spheroid, from multiple experiments. *MFI* mean fluorescence intensity, the data represent mean ± S.E.M. Significance was calculated using unpaired parametric *t* test (***p < 0.001; **p < 0.01). There was 35.3% reduction in UTMC-induced increase in TRD uptake upon GsMTx4 treatment.

Microbubble cavitation-induced shear stress has been shown to activate mechanosensitive channels (such as Piezo1, TRPV4, TRPC1), leading to increased Ca^2+^ influx^29–33^. Here, we hypothesized that in our BBB model, UTMC causes Ca^2+^ influx via mechanosensitive channels. We used a live-cell imaging setup with a cone housing designed in-house and a 0.5-inch immersion transducer (Fig. 4A). First, we confirmed the occurrence of sonoporation in this setup using cell-impermeable propidium iodide (PI) uptake (Supplementary Video S2). UTMC treatment (frequency 1 MHz; PNP 250 kPa; pulse length 10 µs; pulse interval 10 ms; treatment duration 10 s) of spheroids preloaded with the Ca^2+^ indicator Fluo 4-AM increased Ca^2+^ influx (Fig. 4B-Vehicle, Supplementary Video S3). When the spheroids were treated with 1 µM GsMTx4 for 30 min, there was a significant reduction in Ca^2+^ influx (10.8% reduction, *p* < 0.05) (Fig. 4B-GsMTx4, area under the curve shown in Fig. 4C), confirming the involvement of mechanosensitive channels in UTMC-induced Ca^2+^ influx. Furthermore, treatment of spheroids with GsMTx4 resulted in a 35.3% reduction in UTMC-induced penetration of TRD into the spheroid (Fig. 4D,E), indicating that Ca^2+^ influx via mechanosensitive channels regulates UTMC-induced hyperpermeability.

### UTMC increases nitric oxide production, which is necessary for hyperpermeability

**Figure 5:**
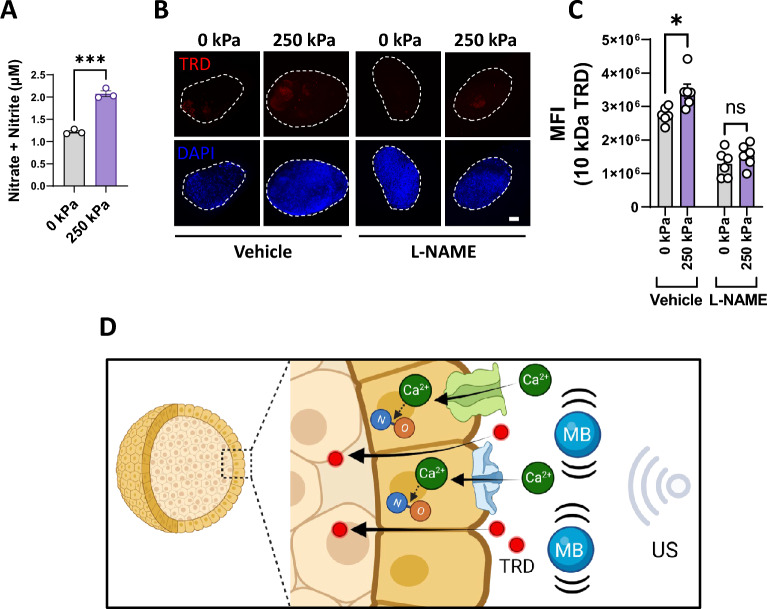
UTMC causes increased nitric oxide production, which is necessary for hyperpermeability. (**A**) Amounts of total nitrate + nitrites in the media (indirect measure of NO) upon UTMC. (**B**) Representative images showing TRD uptake in spheroids treated with L-NAME (1 mM for 1 h). All images are maximum intensity Z-projections of the optical z-stacks. Nuclei are counterstained with DAPI (blue). Scale bar = 100 µm. (**C**) Quantitation of TRD uptake. Each dot represents one spheroid, from multiple experiments. The data represent mean ± S.E.M. Significance was calculated using unpaired parametric t-test (ns: not significant, *p < 0.05, ***p < 0.001). (**D**) Schematic summarizing the working paradigm based on this study’s data (*TRD* Texas red dextran, *MB* microbubble, *US* ultrasound, *NO* nitric oxide).

Nitric oxide (NO) generated by activated endothelial nitric oxide synthase (eNOS) has been shown to regulate endothelial barrier function by modulating VE-cadherin^34^. As Ca^2+^ influx can increase NO production^35–37^, we hypothesized that UTMC-induced Ca^2+^ influx regulates hyperpermeability via the eNOS pathway. We measured nitrites and nitrates as an indirect indicator of NO production, as NO rapidly oxidizes to form these compounds^38^. When spheroids were treated with UTMC, there was a 70.3% increase (*p* < 0.001) in nitrate + nitrites in the media (Fig. 5A). The increase in NO was dependent on UTMC-induced eNOS activation (Supplementary Fig. S3). While vehicle controls showed a significant increase in UTMC-induced TRD uptake (*p* < 0.05), treatment of spheroids with the eNOS inhibitor L-NAME (1 mM for 1 h) abrogated the UTMC-induced effect (*p* = 0.27) (Fig. 5B,C), indicating that the eNOS pathway is involved in regulating UTMC-induced hyperpermeability in our BBB spheroid model. The results of inhibiting eNOS with L-NAME demonstrated a significantly greater reduction in UTMC-induced hyperpermeability when compared to the inhibition of Ca^2+^ influx. The incomplete inhibition of Ca^2+^ influx using GsMTx4 could potentially explain this observed difference. These data demonstrate that our model is suitable for investigating drug permeability dynamics and the pathways that regulate BBB permeability.

## Discussion

Over the past three decades, there has been a substantial increase in the prevalence of neurological disorders, including Alzheimer's disease, Parkinson's disease, and dementia^39^, which is exacerbated by the growth of the aging population. Additionally, there has been a significant rise in the incidence of cancer metastases to the brain^40^. Despite research aimed at developing new drugs to treat these conditions, the presence of the blood‒brain barrier (BBB) remains a significant obstacle to the clinical application of new therapeutics. Consequently, there is a demand for innovative methodologies that can improve the uptake of existing and new BBB-impermeable drugs. As an alternative to expensive animal models, this study presents a novel approach in which we have developed a 3D, multicellular human brain spheroid model that closely mimics the BBB both structurally and functionally. Unlike the physiological cylindrical geometry of the microcirculation wherein a microbubble would be boundaried by the vessel wall, our BBB model is spherical, and the microbubbles are surrounded by culture medium, which might have influenced some of the responses we observed. However, despite this dissimilarity in physical set-up, our spherical BBB model has provided valuable insights into certain aspects of BBB behavior and drug permeability, contributing to our understanding of the essential functions of the BBB and its response to UTMC. Further research and modeling refinements may help bridge this gap.

UTMC is a novel technology that has garnered significant interest for its potential to transiently open the BBB and improve drug delivery^41^. In our spheroid model, we show that UTMC enhances drug permeability. Further experiments using chemical inhibitors indicated that UTMC-induced Ca^2+^ influx and nitric oxide production regulate hyperpermeability. With a functional BBB at the periphery and a complex assembly of other brain cell types at the core, our 3D brain spheroid model provides a system for studying pathways regulating BBB permeability.

Previous studies have successfully generated complex brain organoids using iPSCs, closely mimicking in vivo physiology^42–45^. While these organoids offer numerous advantages over 2D models, reproducing intricate brain structures and exhibiting prolonged viability, their cost and labor-intensive generation can be prohibitive. Alternatively, some studies have utilized primary cells and cell lines to form aggregates or spheroids, demonstrating BBB properties^16,19,46^. Although these models are quicker to generate and useful for studying permeability and bioeffects, they may lack the sophisticated cellular organization found in organoids, and immortalization of endothelial cells can lead to the loss of specific properties^47^. Our model represents a ‘hybrid’ system that combines hiPSC-derived cells (neurons, astrocytes) with primary human brain-derived cells (HBMECs, pericytes, and microglia). This approach is comparatively easier and faster to generate, similar to a model reported earlier by Nzou et al.^20^. This hybrid system strikes a balance between complexity and feasibility, making it an attractive platform for studying drug permeability and bioeffects in the context of the BBB.

Our model combines intricate organization with relative ease of establishment. The key feature of our model is the presence of densely packed endothelial cells on the surface, that serves as a functional blood–brain barrier (BBB). While the distribution of neurons, astrocytes, and microglia did not show a consistent pattern, staining astrocytic end-feet with Aqp4 revealed interactions between astrocytes and cells on the periphery (data not presented). This suggests that our model holds promise for investigating the bioeffects on astrocytes. In this study, we imaged cells at depths of up to 200 µm, and we are currently optimizing methods for comprehensive staining and visualization throughout the spheroids. This optimization will greatly enhance the potential for imaging-based studies using our model. While some previously reported models^19^ are cost-effective and scalable, they often lack the cell remodeling observed in our model due to the limited time allowed for establishment. In comparison to other previously reported spheroid models that comprised three cell types^16,46^, our model includes neurons and microglia as well, enabling us to study bioeffects of UTMC and other permeability enhancing techniques. We have deliberately avoided using immortalized endothelial cells, as they are known to lose certain critical BBB properties. Additionally, our model demonstrates consistently high cell viability over several weeks, making it valuable for certain long-term experiments.

Microbubbles, due to their buoyancy, tend to float up in the media and move away from the cells, which can be problematic during experiments. However, we overcame this issue by using shallow media, just enough to cover the spheroids, ensuring a consistent presence of sufficient microbubbles in close proximity to the spheroids throughout our study (Supplementary Video S1). We examined the impact of different acoustic pressures (0, 250, 350, and 500 kPa) on cell viability and permeability (data not shown). Remarkably, even at a relatively low pressure of 250 kPa, we observed a statistically significant increase in 10 kDa TRD uptake without causing significant cell death (Fig. 3C–E).

Using PI, we detected sonoporation occurring in the cells on the surface (Supplementary Video S2). Surprisingly, we found some sonoporated cells as deep as 100 µm beyond the endothelial layer (data not shown). This intriguing finding warrants further exploration to determine if it represents an example of ‘remote sonoporation', or if it is attributed to the presence of microbubbles that could penetrate deeper into the spheroids, potentially due to the permeated barrier. Although this finding was not explored further in the present study, our model allows us to investigate the bioeffects of UTMC not only on endothelial cells but also on cells beyond the endothelial barrier.

Previous studies, mostly conducted with 2D systems, have demonstrated that microbubble cavitation-induced shear stress enhances Ca^2+^ influx by activating mechanosensitive channels^30–33^. However, documenting Ca^2+^ influx in a 3D setting poses unique challenges. We employed live-cell imaging and recorded Fluo 4-AM fluorescence within cells on the surface (Fig. 4B, Supplementary Video S3). In the 3D environment, numerous cells were located at different planes, resulting in some being out of focus during imaging. We used GsMTx4 to validate the role of mechanosensitive channels in UTMC-induced Ca^2+^ influx. As GsMTx4 inhibits mechanosensitive channels from both the Piezo and TRP channel families, conducting additional studies employing specific inhibitors or siRNAs targeting individual channels could provide valuable insights.

Studies have demonstrated that Ca^2+^ influx can lead to an increase in nitric oxide (NO) production^35–37^. Additionally, eNOS and NO have been identified as crucial components in the regulation of microvascular hyperpermeability^48^. In our study, we employed two different indirect methods to measure nitric oxide and confirmed that UTMC leads to increased NO production (Fig. 5A and Supplementary Fig. S3). Inhibition of eNOS resulted in a reduction in UTMC-induced hyperpermeability (Fig. 5B,C), providing compelling evidence for the involvement of the eNOS pathway in regulating permeability in our model (Fig. 5D). Using high-magnification confocal microscopy on our 3D spheroid model should allow us to further assess the impact of activated eNOS on adherens and tight junction proteins and their role in mediating UTMC-induced BBB hyperpermeability. This approach promises to yield valuable insights into the underlying mechanisms governing vascular permeability in response to shear stress.

UTMC represents a significant innovation, as the BBB poses a major obstacle for the delivery of various therapeutics to the central nervous system^4^. UTMC has shown great promise, particularly in addressing drug delivery challenges in disorders such as Alzheimer's disease (AD). AD is marked by the presence of amyloid-beta plaques and tau protein tangles in the brain. UTMC has demonstrated its potential in delivering drugs and antibodies to reduce these plaques^49–53^. Intriguingly, studies have shown that even without drugs, the use of ultrasound with microbubbles alone reduces amyloid plaques and improves cognitive abilities in mouse models of AD^12,31,54,55^. Our 3D spheroid model incorporates neurons and astrocytes derived from human iPSCs. By substituting these cells with those derived from AD patient-specific iPSCs, we can recreate the unique genetic and molecular characteristics of individual patients, which would provide a closer representation of the in vivo conditions^56,57^, allowing for more physiologically relevant experiments. Through drug testing on these patient-specific spheroids, we can anticipate individual responses to medications, detect potential side effects, and fine-tune treatment strategies for enhanced effectiveness.

## Conclusions

The growing prevalence of neurological disorders defines an urgent need to develop an ideal in vitro model that can facilitate the design of novel technologies for enhanced drug delivery across the blood‒brain barrier (BBB). Here, we introduce a 3D spheroid model that incorporates five distinct brain cell types, mimicking the BBB, both structurally and functionally. This model not only allows us to investigate the pathways involved in regulating hyperpermeability but also provides a valuable platform to study the effects of permeability-enhancing technologies on different brain cell types. Importantly, using patient-specific iPSCs to derive neurons and astrocytes, this model may enable us to replicate the distinctive genetic and molecular traits of individual patients, leading to a more accurate representation of in vivo conditions.

## Methods

### Cells and media

The neural precursor cells (NPCs) derived^58^ from human induced pluripotent stem cell (hiPSC) line SC0000020 (subclone SF) (generated at RUCDR Infinite Biologics, Piscataway, NJ, USA) were maintained in STEMdiff Neural Progenitor Medium (05833, STEMCELL Technologies, Vancouver, BC, Canada). The iPSCs were generated using fibroblasts derived from skin biopsies collected from control individuals. The University of Pittsburgh Institutional Review Board approved the study, and subjects signed written informed consent prior to participation. Astrocytes were derived from these NPCs using a STEMdiff Astrocyte Differentiation kit (100-0013, STEMCELL Technologies) and were maintained in Astrocyte Medium (1801, ScienCell, San Diego, CA, USA). Microglia (C1110, FUJIFILM Cellular Dynamics, Madison, WI, USA), human brain microvascular endothelial cells (HBMECs) (ACBRI376, CellSystems, Kirkland, WA, USA), and human brain vascular pericytes (1200, ScienCell) were commercially procured and maintained in respective media (iCell Microglia Medium, R1204, FUJIFILM Cellular Dynamics; Complete Classic Medium, 4Z0-500, CellSystems; and Pericyte Medium, 1201, ScienCell). Cortical Organoid Differentiation Medium, phase I (CODM-I) contained Gibco Dulbecco's Modified Eagle Medium, Nutrient Mixture F-12 (11330-032, Thermo Fisher Scientific, Waltham, MA, USA), Gibco Neurobasal Medium (21103-049, Thermo Fisher Scientific) with a volume ratio of 1:1, 0.5 × N2 supplement (17502-048, Thermo Fisher Scientific), 0.5 × MEM Nonessential Amino Acid supplement (25-025-CI, Corning, NY, USA), 1 × B27 supplement minus Vitamin A (12587-010, Thermo Fisher Scientific), 1 × Glutamax (35050-061, Thermo Fisher Scientific), 1 × antibiotic/antimycotic (15240-062, Anti/Anti, Thermo Fisher Scientific), and 2.5 µg/mL Insulin solution (I9278, Millipore Sigma, St. Louis, MO, USA). Co-Culture Medium (CCM) contained a 1:3:1 volume ratio of Astrocyte Medium, Neurobasal medium (supplemented with 1 × Anti/Anti, 1 × Glutamax, 1 × B27 with Vitamin A (17504-044, Thermo Fisher Scientific), BDNF 10 µg/mL (78005, STEMCELL Technologies), and Complete Classic Medium.

### 3D spheroid generation and culture

A total of 20,000 NPCs, 15,000 astrocytes, and 5000 microglia were mixed in CODM-I and added to each well of a 96-well U-shaped bottom microplate (174925, Thermo Fisher Scientific) (refer to Fig. 1 for the schematic). The plate was then quickly spun down at 2,500 rpm for 30 s and placed in the incubator under standard conditions (37 °C, 5% CO_2_, and 100% humidity). Media change was initiated 3 days after seeding, and half of the media was replaced every other day for 2 weeks. On the first day of week 3, CCM was used to resuspend HBMECs and pericytes. Subsequently, most of the media from the original plate, which contained a mixture of NPCs, astrocytes, and microglia, was discarded and replaced with new medium containing 32,000 HBMECs and 16,000 pericytes per well. The plate was again quickly spun down at 2500 rpm for 30 s and kept in the incubator under standard conditions. Media change was initiated 3 days after seeding, with half of the media replaced every other day until merging of the spheroids was noticeable, which took approximately 2 weeks. Finally, the spheroids were transferred to a 10 cm dish with 15 mL of medium using Axygen® 200 µL wide-bore tips (T-205-WB-C-R-S, Corning) and placed on an orbital shaker in the incubator at 70 rpm. Media was changed every 3rd day until the spheroids were used for experiments. The spheroids that were compact and spherical with well-defined boundary, exhibiting minimal irregularities were picked for the experiments.

### Microbubble generation

Lipid microbubbles were generated as previously described^9^ with slight modifications. Briefly, a mixture of Polyoxyethylene (40) stearate (P3440, Millipore Sigma), DSPC (1,2-distearol-sn-glycerol-3-phosphocholine) (850365C, Avanti Polar Lipids, Alabaster, AL, USA) and DSPE-mPEG 2000 (1,2-distearoyl-sn-glycero-3-phosphoethanolamine-N-[methoxy (polyethylene glycol)-2000] (ammonium salt) (880120C, Avanti Polar Lipids), dissolved in chloroform (1:2:1 ratio, w/w/w), was evaporated using argon gas for 15 min at room temperature (RT), followed by vacuum drying overnight. The dried lipids were dissolved in saline with brief sonication, with a final lipid concentration of 10 mg/mL. The lipid dispersion was sonicated using a Misonix XL2020 Sonicator Ultrasonic Processor XL (Bioventus, Farmingdale, NY, USA) at a power level of 5.25 in the presence of perfluorobutane (PFB) gas (FluoroMed, Round Rock, TX, USA) for 75 s. Then, the microbubbles were washed twice with 20 mL of saline by incubation at RT for 60 min after each wash. The microbubbles were then resuspended in saline, aliquoted in vials with PFB-filled headspace and stored at 4 °C until use. This method yielded microbubbles with a concentration of approximately 1 × 10^9^/mL and a mean diameter of ~ 3 µm (Fig. 3A), as assessed with a Coulter counter (Multisizer 4e, Beckman Coulter, Indianapolis, IN, USA) with a 30 μm aperture tube.

### Ultrasound setup

For US treatment, the spheroids were placed in shallow media containing 1 × 10^6^ microbubbles per well in a multiwell plate (refer to Fig. 3B) and kept in a custom water tank that housed a 1-inch single-element transducer (model: A302S-SU, frequency 1 MHz, Olympus NDT, MA, USA). Using a Sous Vide machine, the water temperature was maintained at 37 °C. An US pulse was generated (frequency 1 MHz; PNP 250 kPa; pulse length 10 µs; pulse interval 10 ms; treatment duration 10 s) using an arbitrary function generator (AFG3252, Tektronix, Beaverton, OR, USA) and amplified by a gated RF power amplifier (model 250A250AM8, Amplifier Research, Bothell, WA, USA). The US system was calibrated in a separate water tank using a hydrophone (HGL-0200, Onda Corp, Sunnyvale, CA, USA).

### Cell viability assay

To confirm that the US parameters were not deleterious to the spheroids, we evaluated cell viability using Calcein-AM (C3100MP, Thermo Fisher Scientific) and SYTOX Red (S34859, Thermo Fisher Scientific) dyes. In the presence of microbubbles, spheroids were treated with 0 kPa or 250 kPa US and moved to wells containing 2 µg/mL Hoechst 33342 (H3570, Fisher Scientific), 1 µM Calcein-AM and 5 nM SYTOX Red and incubated for 15 min. The spheroids were washed with PBS, imaged using a Nikon A1 microscope (Nikon, Tokyo, Japan) and analyzed with Fiji. At least 3 spheroids per condition were tested.

### Texas red dextran permeability

To assess the effect of UTMC on permeability, we assessed the amounts of 10 kDa TRD (D1863, Thermo Fisher Scientific) uptake into the spheroids. Spheroids were placed in a multiwell plate containing shallow media with 1 × 10^6^ microbubbles and incubated for 5 min. The presence of microbubbles on the surface of the spheroids was confirmed under a brightfield microscope. The spheroids were then subjected to ultrasound (details provided under ultrasound setup), washed immediately with PBS, and transferred to a well containing 100 µg/mL TRD. The spheroids were then incubated at 37 °C for 15 min, washed with PBS, and fixed in 2% paraformaldehyde (PFA) (50-980-487, Fisher Scientific) for 30 min at RT. The spheroids were washed again with PBS and placed in a well containing DAPI (D9542, Millipore Sigma) for 15 min, followed by another PBS wash. Each spheroid was then moved to an uncoated µ-Slide (81501, Ibidi, Fitchburg, WI, USA) well containing Dako mounting medium (S302380-2, Agilent, Santa Clara, CA, USA) and imaged using a Nikon A1 confocal microscope. To quantify TRD uptake, the fluorescence from the spheroid surface to up to 20 µm was excluded by measuring the intensity from 20 µm to 200 µm in a region of interest (ROI) of 300 µm diameter (Supplementary Fig. S1) so that any nonspecifically adsorbed fluorescence and artifacts at the surface were avoided. The raw integrated density values within the ROI after background subtraction were used to plot the graphs.

### Functionality assays

To check if the spheroids functionally resembled the BBB, they were treated with histamine (H7125-1G, Millipore Sigma) for 15 min, which is known to induce BBB hyperpermeability^20–22^. After the treatment, the spheroids were incubated with TRD for 15 min, washed and fixed in 2% PFA for 30 min at RT. The spheroids were DAPI stained, washed and moved to a µ-slide containing mounting medium and imaged 20 µm to 200 µm depth using a Nikon A1 confocal microscope (Supplementary Fig. S1).

### Immunostaining and confocal imaging

Immunofluorescence (IF) staining of the spheroids was carried out in Eppendorf tubes. The spheroids were fixed with 2% (when IF was preceded with TRD permeability) or 4% PFA for 60 min at RT. Spheroids were then permeabilized and blocked in 5% normal goat serum solution (S-1000-20, Vector labs, Newark, CA, USA) containing 0.2% Triton-X-100 (X100, Millipore Sigma) for 60 min. Spheroids were then washed with PBS and incubated in primary antibodies dissolved in blocking solution overnight at 4 °C (refer to Supplementary Table S1 for the complete antibody information). The next day, the spheroids were washed multiple times with PBS and incubated overnight at 4 °C in PBB solution [PBS with 0.5% bovine serum albumin (A2153, Millipore Sigma)] containing respective secondary antibodies (refer to Supplementary Table S1) and DAPI. The following day, the spheroids were gently washed with PBB solution multiple times, moved to a µ-slide containing mounting medium.

Imaging was performed using a 4-color Nikon A1 confocal microscope. Nikon Elements AR software was used for image capture and deconvolution. To measure TRD, Calcein-AM, and SYTOX red, z-stack images were taken using a 10 × objective up to 200 µm depth with 5 µm intervals (a total of 41 sections) with Galvano scanning. In each experiment, the aperture, pinhole, digital zoom, and scan speeds were kept constant for all the samples. To image immunostained spheroids, multiple objectives were used (with immersion oil when required). Post-acquisition analysis was carried out using the Fiji application^59^. Before analysis, the images were converted to 8-bit images. To show the immunostained images, Z-projection using Fiji was carried out.

### Calcium imaging

To image calcium flux, the spheroids were loaded with freshly dissolved, cell-permeable 1 µM Fluo 4-AM (F14201, Thermo Fisher Scientific), a green fluorescent, intracellular Ca^2+^ indicator, for 30 min at 37 °C. Then, the spheroids were moved to a glass bottom dish (P50G-0-30-F, MatTek, Ashland, MA, USA) containing media with 1 × 10^6^ microbubbles. A coverslip was placed above the spheroids. The presence of microbubbles on the spheroids was microscopically confirmed. Using an in-house designed cone housing and a water immersion transducer (A303S-SU, 0.5-inch diameter, Olympus NDT), US was delivered (frequency 1 MHz; PNP 250 kPa; pulse length 10 µs; pulse interval 10 ms; treatment duration 10 s), and green fluorescence was measured for up to 1 min (Supplementary Videos S3, 4). The involvement of mechanosensitive channels in UTMC-induced Ca^2+^ influx was confirmed by pretreating spheroids with 1 µM GsMTx4 (STG-100, Alomone labs, Jerusalem, Israel) for 30 min. The occurrence of sonoporation was confirmed by loading spheroids with 120 µM PI (P3566, Thermo Fisher Scientific) in the same setup (Supplementary Video S2). The videos were quantified using Fiji.

### Measuring nitric oxide

To confirm the involvement of endothelial nitric oxide synthase (eNOS) in UTMC-induced hyperpermeability, the spheroids were treated with the eNOS inhibitor 1 mM l-NAME (0665, Tocris Bioscience, Bristol, UK) for 1 h before UTMC treatment. Intracellular nitric oxide (NO) production was measured using a DAF-FM diacetate (DA) assay (D23844, Thermo Fisher Scientific) according to the manufacturer’s protocol. Briefly, the cell-permeant DAF-FM DA is nonfluorescent until it reacts with NO and forms a fluorescent compound (benzotriazole). Immediately after UTMC, the spheroids were transferred to a well containing 5 µM DAF-FM DA and incubated for 60 min at 37 °C. Spheroids were washed, and fresh media was added and incubated for 30 more min before being imaged with a FITC filter (Olympus IX81 microscope and Nikon A1 confocal microscope). The green fluorescence was quantified using Fiji. Extracellular NO was measured with a Nitrate/Nitrite Colorimetric Assay (780001, Cayman Chemical, Ann Arbor, MI, USA) according to the manufacturer’s protocol. Briefly, the spheroids were placed in a glass dish containing media with microbubbles and treated with UTMC. Immediately, the spheroids were transferred to a well in a 12-well plate with 500 µL media and incubated for 60 min. The first step of the assay where nitrates are converted to nitrites using nitrate reductase was carried out for 2 h at RT. The second step, where Griess reagent converts nitrites into deep purple azo compounds, was carried out for 10 min at RT, and the absorbance was read at 595 nm using a DTX 880 Multimode Detector (Beckman Coulter, Indianapolis, IN, USA).

### Statistical analysis

The statistical analysis was performed using GraphPad Prism 9. The data are presented as the mean ± S.E.M. The experiments were repeated at least three times, unless otherwise mentioned. The *p* values were calculated using unpaired parametric *t* tests. The results were considered significant for *p < 0.05, **p < 0.01 and ***p < 0.001.

### Supplementary Information

Supplementary Information.

Supplementary Legends.

Supplementary Video S1.

Supplementary Video S2.

Supplementary Video S3.

Supplementary Video S4.

## Data Availability

All the data used in this study to reach the conclusions are presented in the paper and the Supplementary materials. The datasets are available from the corresponding author upon reasonable request.
