# Supplementary material for: A multicellular brain spheroid model for studying the mechanisms and bioeffects of ultrasound-enhanced drug penetration beyond the blood‒brain barrier
Source: Sci Rep. 2024 Jan 22;14:1909. doi: 10.1038/s41598-023-50203-3 (PMC10803331; doi:10.1038/s41598-023-50203-3)
Supplement: Supplementary file 1 — Supplementary Information. [file 41598_2023_50203_MOESM1_ESM.pdf]

Supplementary Information

A multicellular brain spheroid model for studying the mechanisms and bioeffects of ultrasound-enhanced drug penetration beyond the blood–brain barrier

Anurag N. Paranjape[1,2], Leonardo D'Aiuto[3], Wenxiao Zheng[3,4], Xucai Chen[1,2], and Flordeliza S. Villanueva[1,2 #]

[1]Center for Ultrasound Molecular Imaging and Therapeutics, University of Pittsburgh, Pittsburgh, PA, USA

[2]Department of Medicine, University of Pittsburgh, Pittsburgh, PA, USA

[3]Department of Psychiatry, University of Pittsburgh School of Medicine Western Psychiatric Institute and Clinic, Pittsburgh, PA, USA

[4]Department of Health and Human Development, University of Pittsburgh, Pittsburgh, PA, USA

[#]Corresponding author: villanuevafs@upmc.edu

Supplementary Table. S1. **Antibodies used in the study**.

**Name**	**Dilution**	**Catalog No.**	**Company**
ZO1	1:50	61-7300	Thermo Fisher Scientific
ZO1	1:50	sc-33725	Santa Cruz Biotechnology, Dallas, TX, USA
CD31	1:50	BS-0195R	Bioss Antibodies, Woburn, MA, USA
GFAP	1:50	3670S	Cell signaling technology, Danvers, MA, USA
MAP2	1:50	4542S	Cell signaling technology
NG2	1:50	55027-1-AP	Proteintech, Rosemont, IL, USA
TMEM119	1:50	TMEM119	Proteintech
Phalloidin-iFluor™ 647 Conjugate	1:500	23127	AAT Bioquest, Pleasanton, CA, USA
Goat anti-Mouse IgG- Alexa Fluor™ 488	1:500	A-11001	Thermo Fisher Scientific
Goat anti-Mouse IgG- Alexa Fluor™ 568	1:500	A-11004	Thermo Fisher Scientific
Goat anti-Rabbit IgG- Alexa Fluor™ 488	1:500	A-11008	Thermo Fisher Scientific
Goat anti-Rabbit IgG- Alexa Fluor™ 568	1:500	A-11011	Thermo Fisher Scientific
Goat anti-Rat IgG- Cyanine5	1:500	A-10525	Thermo Fisher Scientific

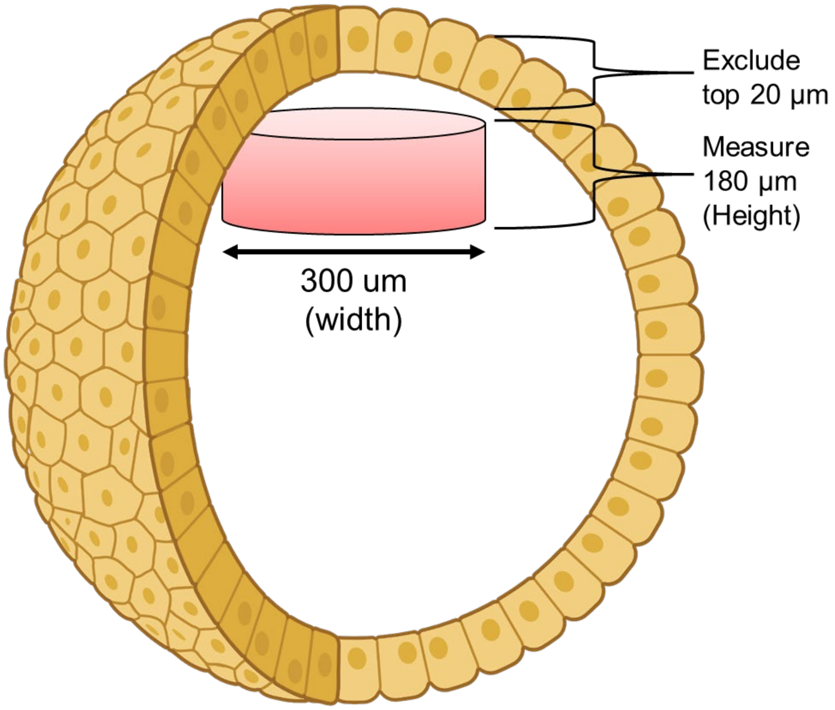

Supplementary Figure S1. **Quantitation of Texas red dextran inside the spheroids.** Schematic (not to scale) illustrating the process of acquiring and quantifying 10 kDa Texas red dextran. To avoid non-specifically adsorbed fluorescence and artifact present at the spheroid surface, the fluorescence signal from the surface to up to 20 µm depth was excluded. Fluorescence from optical sections from 20 µm to 200 µm in a region of interest (ROI) of 300 µm width was used to plot the graphs after background correction.

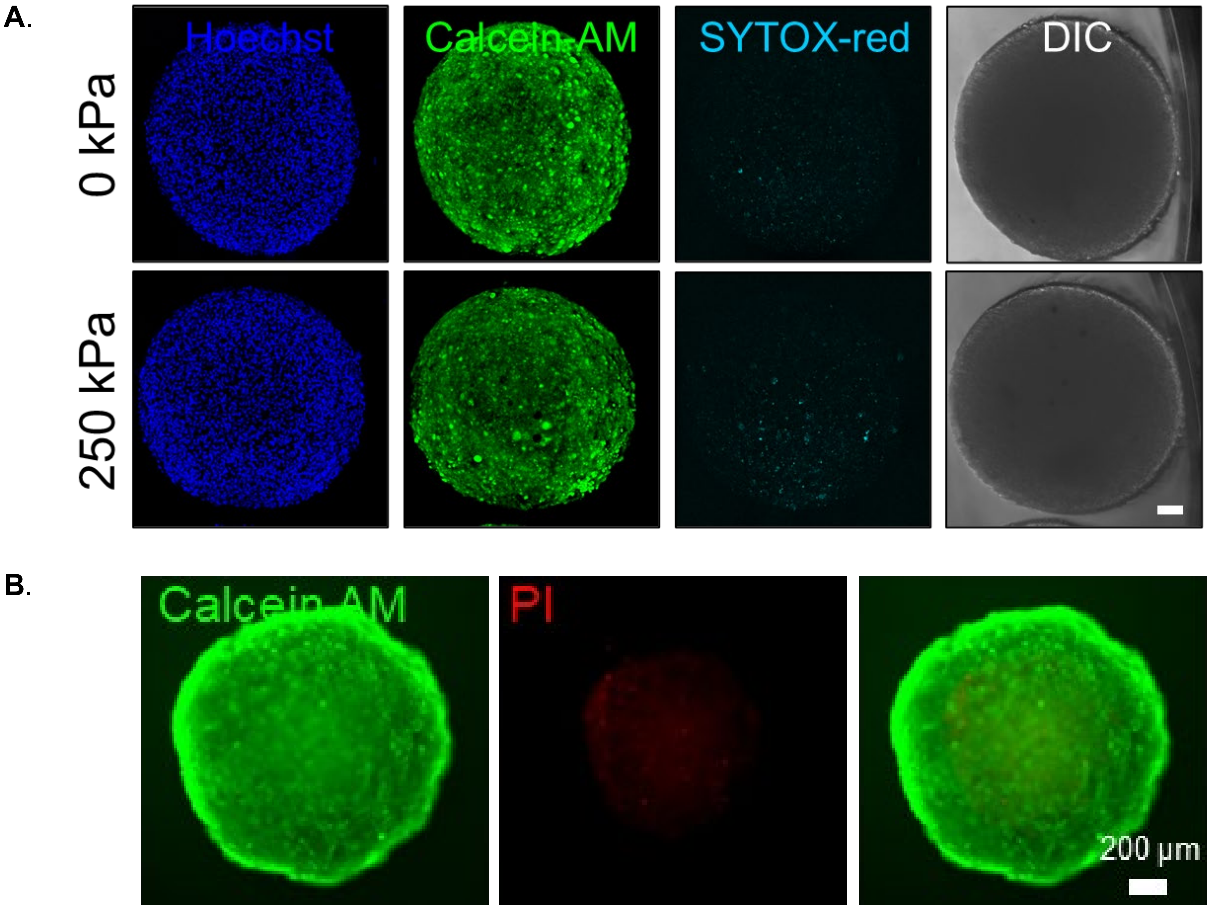

Supplementary Figure S2. **Cell-viability assay. A**. Representative images showing UTMC-treated spheroids stained with Hoechst 33342, Calcein-AM and SYTOX red for 15 minutes. The optical sections were converted to maximum intensity Z-projection and the mean fluorescence intensity (MFI) was plotted (refer to Fig. 3C). Scale bar = 100 µm. **B**. Representative spheroid showing cell viability after 1 hr of UTMC treatment (scale bar = 200 µm).

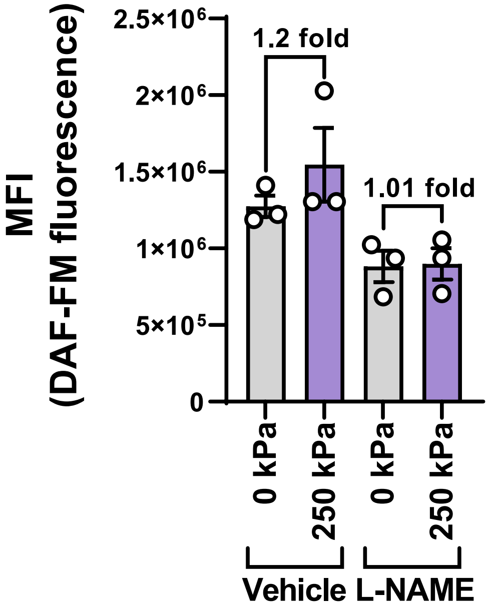

Supplementary Figure. S3. **DAF-FM assay to quantify nitric oxide (NO)**. To assess the UTMC-induced NO generation, DAF-FM assay was performed in the presence of eNOS inhibitor L-NAME. DAF-FM diacetate is a cell-permeable non-fluorescent compound which reacts with NO to form a fluorescent triazole derivative which was measured using microscopy and the green fluorescence was quantified using ImageJ (also refer to Figure. 5). Each dot represents a spheroid, MFI: mean fluorescence intensity. The data represents mean ± S.E.M.

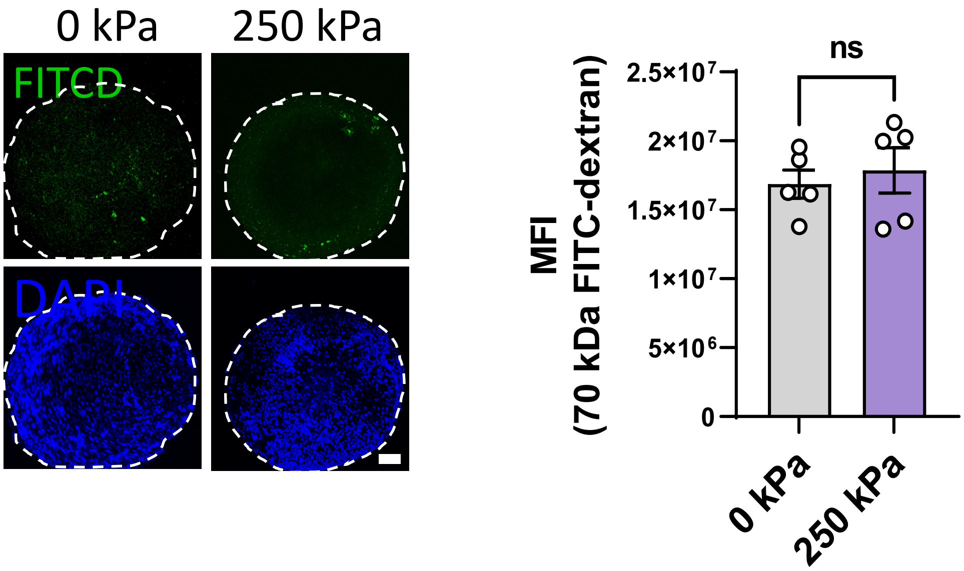

Supplementary Figure S4. **Effect of UTMC on 70 kDa FITC-dextran uptake**. Representative images showing 70 kDa FITC-dextran inside the spheroids. The spheroid boundary is shown with dashed white line. All images are maximum intensity Z-projections of the optical z-stacks. Nuclei were counterstained with DAPI (blue), scale bar = 100 µm. The graph on the right shows quantified dextran uptake. Each dot represents one spheroid, from multiple experiments. MFI: mean fluorescence intensity, the data represent mean ± S.E.M. Significance was calculated using unpaired parametric t-test (ns: not significant).
