# Supplementary material for: A multicellular brain spheroid model for studying the mechanisms and bioeffects of ultrasound-enhanced drug penetration beyond the blood‒brain barrier
Source: Sci Rep. 2024 Jan 22;14:1909. doi: 10.1038/s41598-023-50203-3 (PMC10803331; doi:10.1038/s41598-023-50203-3)
Supplement: Supplementary file 2 — Supplementary Legends. [file 41598_2023_50203_MOESM2_ESM.pdf]

Supplementary Table S1. **Antibodies used in the study**.

Supplementary Figure S1. **Quantitation of Texas red dextran inside the spheroids**. Schematic (not to scale) illustrating the process of acquiring and quantifying 10 kDa Texas red dextran. To avoid non-specifically adsorbed fluorescence and artifact present at the spheroid surface, the fluorescence signal from the surface to up to 20 μm depth was excluded. Fluorescence from optical sections from 20 μm to 200 μm in a region of interest (ROI) of 300 μm width was used to plot the graphs after background correction.

Supplementary Figure S2. **Cell-viability assay**. **A.** Representative images showing UTMC-treated spheroids stained with Hoechst 33342, Calcein-AM and SYTOX red for 15 minutes. The optical sections were converted to maximum intensity Z-projection and the mean fluorescence intensity (MFI) was plotted (refer to Fig. 3C). Scale bar = 100 μm. **B**. Representative spheroid showing cell viability after 1 hr of UTMC treatment (scale bar = 200 μm).

Supplementary Figure. S3. **DAF-FM assay to quantify nitric oxide (NO).** To assess the UTMC-induced NO generation, DAF-FM assay was performed in the presence of eNOS inhibitor L-NAME. DAF-FM diacetate is a cell-permeable non-fluorescent compound which reacts with NO to form a fluorescent triazole derivative, which was imaged using fluorescence microscopy and quantified using Fiji (also refer to Figure. 5). Each dot represents a spheroid, MFI: mean fluorescence intensity. The data represents mean ± S.E.M.

Supplementary Figure S4. **Effect of UTMC on 70 kDa FITC-dextran uptake.** Representative images showing 70 kDa FITC-dextran inside the spheroids. The spheroid boundary is shown with dashed white line. All images are maximum intensity Z-projections of the optical z-stacks. Nuclei were counterstained with DAPI (blue), scale bar = 100 μm. The graph on the right shows quantified dextran uptake. Each dot represents one spheroid, from multiple experiments. MFI: mean fluorescence intensity, the data represent mean ± S.E.M. Significance was calculated using unpaired parametric t-test (ns: not significant).

Supplementary Video S1. **Microbubbles on the spheroid surface**. DIC video of a spheroid showing microbubbles on the spheroid surface. The focus was manually changed using the fine adjustment knob to confirm presence of MBs in different planes of the curved surface.

Supplementary Video S2. **Visualization of sonoporation**. Video showing sonoporation occurring in cells on the surface of a spheroid. The cell-impermeable PI (in red) enters the cell and labels nuclei (white arrows) upon UTMC.

Supplementary Video S3. **Visualization of Ca^{2+} influx using Fluo 4-AM (Vehicle control)**. Video showing Fluo 4-AM fluorescence (green) indicating Ca^{2+} influx upon UTMC in a Vehicle control spheroid.

Supplementary Video S4. **Visualization of Ca^{2+} influx using Fluo 4-AM (GsMTx4-treated)**. Video showing Fluo 4-AM fluorescence (green) indicating Ca^{2+} influx upon UTMC in a GsMTx4-treated spheroid.
